# Involvement of ethylene receptors in the salt tolerance response of *Cucurbita pepo*

**DOI:** 10.1038/s41438-021-00508-z

**Published:** 2021-04-01

**Authors:** Gustavo Cebrián, Jessica Iglesias-Moya, Alicia García, Javier Martínez, Jonathan Romero, José Javier Regalado, Cecilia Martínez, Juan Luis Valenzuela, Manuel Jamilena

**Affiliations:** Department of Biology and Geology, Agri-food Campus of International Excellence (CeiA3) and Research Center CIAMBITAL, University of Almería, 04120 Almería, Spain

**Keywords:** Plant breeding, Plant biotechnology

## Abstract

Abiotic stresses have a negative effect on crop production, affecting both vegetative and reproductive development. Ethylene plays a relevant role in plant response to environmental stresses, but the specific contribution of ethylene biosynthesis and signalling components in the salt stress response differs between Arabidopsis and rice, the two most studied model plants. In this paper, we study the effect of three gain-of-function mutations affecting the ethylene receptors *CpETR1B*, *CpETR1A*, and *CpETR2B* of *Cucurbita pepo* on salt stress response during germination, seedling establishment, and subsequent vegetative growth of plants. The mutations all reduced ethylene sensitivity, but enhanced salt tolerance, during both germination and vegetative growth, demonstrating that the three ethylene receptors play a positive role in salt tolerance. Under salt stress, *etr1b*, *etr1a*, and *etr2b* germinate earlier than WT, and the root and shoot growth rates of both seedlings and plants were less affected in mutant than in WT. The enhanced salt tolerance response of the *etr2b* plants was associated with a reduced accumulation of Na^+^ in shoots and leaves, as well as with a higher accumulation of compatible solutes, including proline and total carbohydrates, and antioxidant compounds, such as anthocyanin. Many membrane monovalent cation transporters, including Na^+^/H^+^ and K^+^/H^+^ exchangers (NHXs), K^+^ efflux antiporters (KEAs), high-affinity K^+^ transporters (HKTs), and K^+^ uptake transporters (KUPs) were also highly upregulated by salt in *etr2b* in comparison with WT. In aggregate, these data indicate that the enhanced salt tolerance of the mutant is led by the induction of genes that exclude Na^+^ in photosynthetic organs, while maintaining K^+^/Na^+^ homoeostasis and osmotic adjustment. If the salt response of *etr* mutants occurs via the ethylene signalling pathway, our data show that ethylene is a negative regulator of salt tolerance during germination and vegetative growth. Nevertheless, the higher upregulation of genes involved in Ca^2+^ signalling (*CpCRCK2A* and *CpCRCK2B*) and ABA biosynthesis (*CpNCED3A* and *CpNCED3B*) in *etr2b* leaves under salt stress likely indicates that the function of ethylene receptors in salt stress response in *C. pepo* can be mediated by Ca^2+^ and ABA signalling pathways.

## Introduction

One of the great challenges facing agriculture today is the development of production systems that mitigate the deleterious effects of climate change, including drought and salinity^1^. In arid and semi-arid areas, soil and water salinity constitute two of the most important abiotic stresses that limit crop production. At present, >1 billion hectares worldwide are affected by soil salinity^2^.

Crop development and performance is severely affected by salinity. The primary effects of salinity are very similar to those caused by drought. A high concentration of salt in the soil reduces the plant’s ability to absorb water, known as the osmotic effect due to salinity. This not only leads to reduced absorption of essential elements, such as K^+^, Ca^2+^ and NO_3_^−^, but also a toxic accumulation of Na^+^ and Cl^−^ in aerial parts of the plant^3^. The accumulation of salt in leaf cells inhibits cell expansion and photosynthetic activity, which ultimately leads to a reduction in crop yield^4^.

The entrance and the perception of Na^+^ in roots are little known processes. Sodium can enter the root through non-selective cation channels (NSCCs)^5^, although extracellular cation receptors, such as MONOCATION INDUCED [Ca^2+^] and INCREASES 1 (MOCA1), have been detected, which are capable of sensing sodium and other cations, as well as promoting the influx of Ca^2+^ into the cell^6,7^. The perception of the stress signal triggers a secondary signalling by reactive oxygen species (ROS) and abscisic acid (ABA), which also regulate the intracellular level of Ca^2+^. The cytosolic calcium activates phosphorylation cascades of Ca^2+^-dependent proteins or calcium sensors, including calmodulins (CaM), CaM-like (CML) and calcineurin B-like proteins (CBL), which leads to regulation of stress response genes^8,9^.

To deal with salinity, plants have implemented three general mechanisms that improve plant tolerance to salt stress: (i) restoration of ion homoeostasis (Na^+^/K^+^ homoeostasis); (ii) restoration of osmotic homoeostasis; and (iii) prevention and repair of cell damage. Ionic homoeostasis mediated by membrane ion transporters constitutes the main response mechanism against salt stress. Plasma membrane Na^+^/H^+^ antiporters, such SALT OVERLY SENSITIVE 1 (SOS1/AtNHX7) and AtNHX8, extrude Na^+^ into the extracellular medium in response to an increase in intracellular Ca^2+^. HIGH-AFFINITY K^+^ TRANSPORTER1-like (HTK1-like) also has a strong affinity for Na^+^^9^, which excludes the translocation of Na^+^^10,11^. Tonoplast Na^+^/H^+^ antiporters, such as Na^+^/H^+^ EXCHANGER 1–4 of Arabidopsis (NHX1-4), are also activated by Ca^2+^, transporting Na^+^ (and K^+^) into the vacuole^12–14^, reducing toxic Na^+^ in the cytoplasm, and decreasing the osmotic potential of the cell. The overexpression of both plasma membrane and tonoplast antiporters results in a greater tolerance to salinity in a wide range of plant species^15–17^. K^+^ transporters, including the high-affinity transporter family HAK/KT/KUP, the HKT family of high-affinity K^+^ transporters and the KEA family of K^+^ efflux antiporters, are also involved in salt tolerance by maintaining K^+^/Na^+^ homoeostasis^10,17^. To restore osmotic homoeostasis and cell volume and turgor, salt also activates the production of compatible solutes or osmolytes, including proline, sugar alcohols, sorbitol and anthocyanins, among others^18,19^. These osmolytes also function as protectors of membranes and proteins by reducing oxidative damage^20,21^.

The phytohormones ABA and ethylene play key roles in the defensive response of plants against abiotic stresses^7,22^. ABA is a positive regulator of plant defensive response. Under both salinity and water deficit, plants induce the production of ABA biosynthesis genes, such as *NINE-CIS-EPOXYCAROTENOID DIOXYGENASES* (*NCEDs*) and A*BA DEFICIENTS* (*ABAs*). ABA is then perceived by the ABA receptors PYRABACTIN RESISTANCE/PYRABACTIN RESISTANCE LIKE (PYR/PYL), which induce phosphorylation activity of the ABA-dependent SUCROSE NON-FERMENTING RELATED PROTEIN KINASES (SnRKs) family, and the activation of the ABA-dependent transcriptional network involved in ionic and osmotic adjustments in response to salt stress^7,23^.

The function of ethylene in salt stress response is, however, more controversial^24^. It is generally presumed that ethylene improves the response of plants to salt stress^25^. However, other authors supported a negative role of ethylene during salt stress, at least in certain growth stages in which its induction can activate oxidative stress and leaf senescence^26^. In Arabidopsis, ethylene positively regulates salinity response, and both ethylene biosynthesis and signalling genes are required for salt tolerance^27,28^. The biosynthesis *ACS* and *ACO* genes in Arabidopsis are induced under salinity conditions, but certain individual members can play a negative role in salt tolerance^29–31^. The ethylene signalling elements also participate in the response of plant to salt stress, but their functions are also unclear^24^. In Arabidopsis, the positive elements of the ethylene response are generally upregulated in response to salt and are positive regulators of salt tolerance; whereas, negative elements are downregulated by salt and are considered to be negative regulators of salt tolerance^24^. In contrast, orthologous ethylene positive signalling genes, including *MHZ7/OsEIN2*, *MHZ6/OSEIL1* and *OsEIL2*, have an opposite function in rice, since their suppression produces salinity tolerance, while their individual overexpression enhances salt sensitivity^32^.

The five ethylene receptors of Arabidopsis, ETR1, ERS1, ETR2, ERS2 and EIN4, are negative regulators of the ethylene signal pathway, but play a contrasting role in salt tolerance. They possess highly similar amino acid sequences and domain structures. The ethylene binding property of all of the receptors resides in three or four N-terminal transmembrane helices that are located within the membrane of the endoplasmic reticulum^33,34^. These N domains are connected by a GAF domain to a C-terminal His protein kinase domain that is positioned in the cytoplasm^33,34^. ETR1, ETR2, and EIN4 have an additional C-terminal receiver domain^34^. Both gain-of-function and loss-of-function mutants have been described for the five Arabidopsis ethylene receptor genes. Dominant gain-of-function mutations in a single receptor gene lead to ethylene insensitivity; whereas, recessive loss-of-function mutations confer little or no phenotype, but the combination of two or three loss-of-function ethylene receptor mutations confers constitutive ethylene responses^35^. The function of the five Arabidopsis ethylene receptor genes in salt tolerance has been investigated in loss-of-function mutants during germination, finding that ETR1 and EIN4 inhibit, while ETR2 stimulates and ERS1 and ERS2 have no effect on, seed germination under salt stress^36–38^. These contrasting roles do not appear to require an ethylene canonical signalling pathway, but occur by regulating ABA signal transduction^24,25,36,39^. Silencing of alfalfa *MsETR2* abolishes ethylene-triggered tolerance to salt stress, indicating that this ethylene receptor is a positive regulator of salt tolerance in alfalfa^40^.

Recently, García et al.^41^ isolated four *Cucurbita pepo* mutants*, etr1a, etr1a-1, etr1b* and *etr2b*, all exhibiting a reduced response to ethylene, as well as concomitant changes in developmental traits regulated by ethylene^41–43^. The four mutations affected sex determination in this monoecious species, as well as female fertility. They convert female into male or female-sterile hermaphrodite flowers, which prevents self-fertilisation of homozygous mutant plants, and forces the maintenance of mutations in segregating populations^42,43^. The duplicated genome of *C. pepo*^44^ contains six ethylene receptor genes, two paralogs for either *ETR1* (*CpETR1A* and *CpETR1B*), *ERS1* (*CpERS1A* and *CpERS1B*) and *ETR2* (*CpETR2A* and *CpETR2B*), and the identified mutations affect three of the ethylene receptor genes. *etr1a-1* and *etr1a* are A95V and P36L amino acid exchanges in the first and third transmembrane helix of CpETR1A, respectively, *etr1b* is a T94I amino acid exchange in the third transmembrane helix of CpETR1B, and *etr2b* is an E340K amino acid exchange in the coiled-coil domain between the GAF and histidine-kinase domains of CpETR2B^42,43^.

In this paper, we investigated the response of *etr1b*, *etr1a*, and *etr2b* gain-of-function mutants to salt stress during germination, seedling establishment, and subsequent vegetative growth. Since the three mutants showed enhanced salt tolerance response during all studied developmental stages and reduced content of Na^+^ in photosynthetic organs, we also analysed the molecular mechanisms involved in the enhanced salt tolerance of the *Cucurbita etr* mutants, including accumulation of osmoprotectants and activation of gene networks involved in the biosynthesis of ABA, Ca^2+^ signalling elements, and Na^+^ and K^+^ membrane transporters reducing the accumulation of toxic Na^+^ in shoots and leaves.

## Results

### Tolerance of *etr1b*, *etr1a*, and *etr2b* to salt stress during germination and early stages of seedling development

**Fig. 1:**
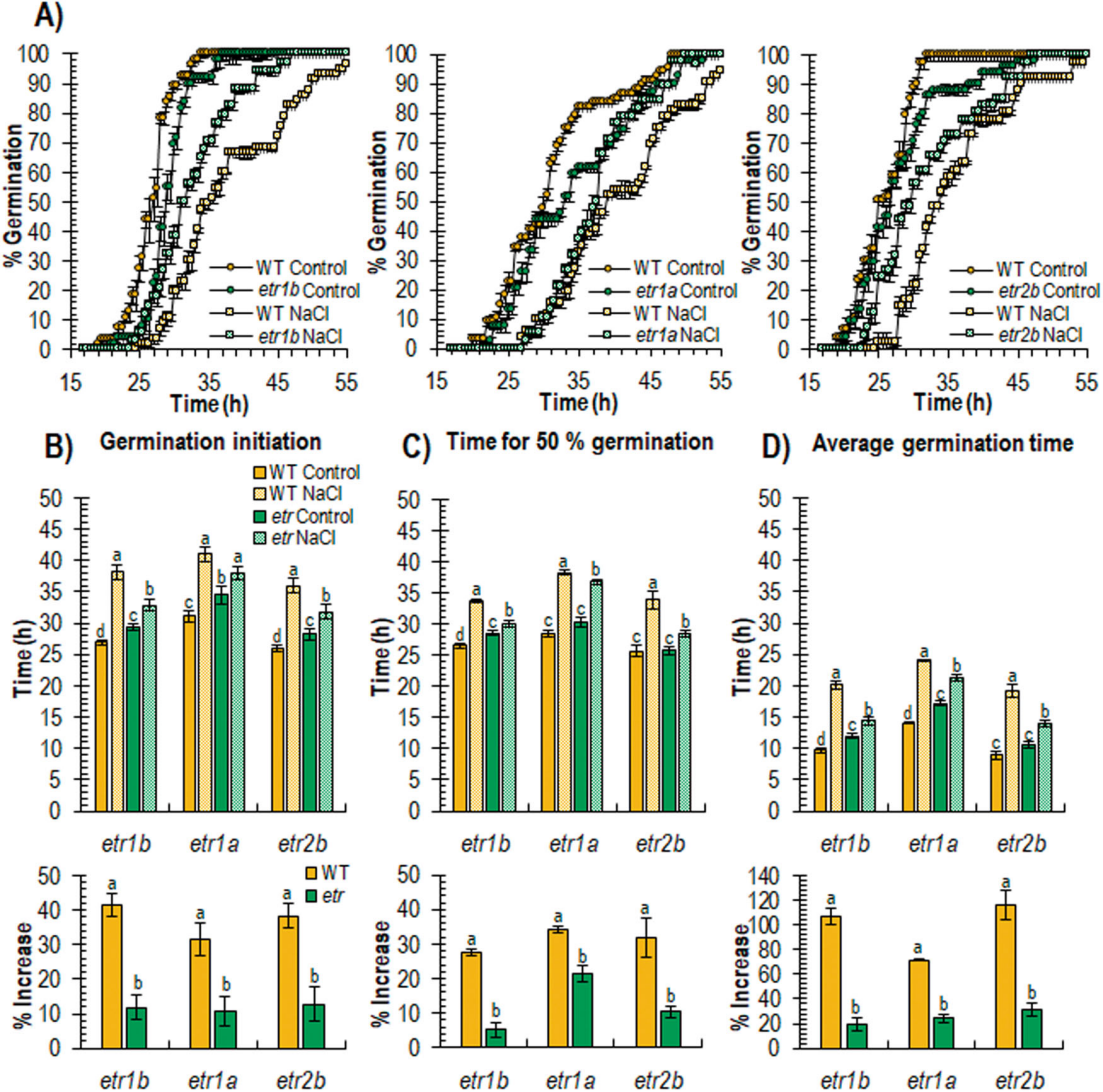
Effect of salt stress on germination parameters of WT and *etr1b*, *etr1a*, and *etr2b*. **A** Germination rates of WT and *etr1b, etr1a*, and *etr2b* ethylene receptor mutants under control and NaCl conditions. The percentage of germination was analysed every 2â€‰h at the indicated time points. The data represent means of three independent replicates with at least 50 seeds counted per replicate. **B**, **C**, and **D** Effect of NaCl stress treatment on germination initiation, time at which 50% of seed is germinated, and average germination time. The bottom graphs show the percentage of increase of each parameter in response to salt stress in WT and mutant plants with respect to plants of the same genotype grown under control conditions. Means were obtained from four independent replicates with at least 50 seeds per replicate. Different letters indicate statistically significant differences (*P*â€‰<â€‰0.05) between samples

To determine the ability of *etr* mutants to germinate in the presence of NaCl, WT and *etr1b*, *etr1a*, and *etr2b* mutant seeds were germinated in both water and 100 mM of NaCl up to 55 h, recording the initiation of seed germination every 2 h. The results are shown in Fig. 1. In water, both WT and the three *etr* mutants showed a similar germination rate, although the mutant seed was slightly delayed with respect to WT (Fig. 1A). The NaCl treatment delayed germination of both WT and *etrs*, but the delayed time was much higher in the WT, meaning that the three *etr* mutants germinated faster than WT under salt stress.

The salt treatment affected WT and mutant seed differently for different germination parameters, including germination initiation, average time for 50% germination, and average germination time (Fig. 1B, D). In water, the assessment of the three germination parameters in *etr* seeds was highly similar to that of WT. Under salt stress, however, there was a significant increase in germination initiation, 50% of germination and average germination time in WT and mutant seeds, but the percentage of increase of these three parameters in NaCl with respect to water was significantly lower in the three mutants compared with their corresponding WT genotypes (Fig. 1B). Taken together, the data revealed that the three *etr* mutants are all more tolerant to salt stress than their corresponding WT during germination.

**Fig. 2:**
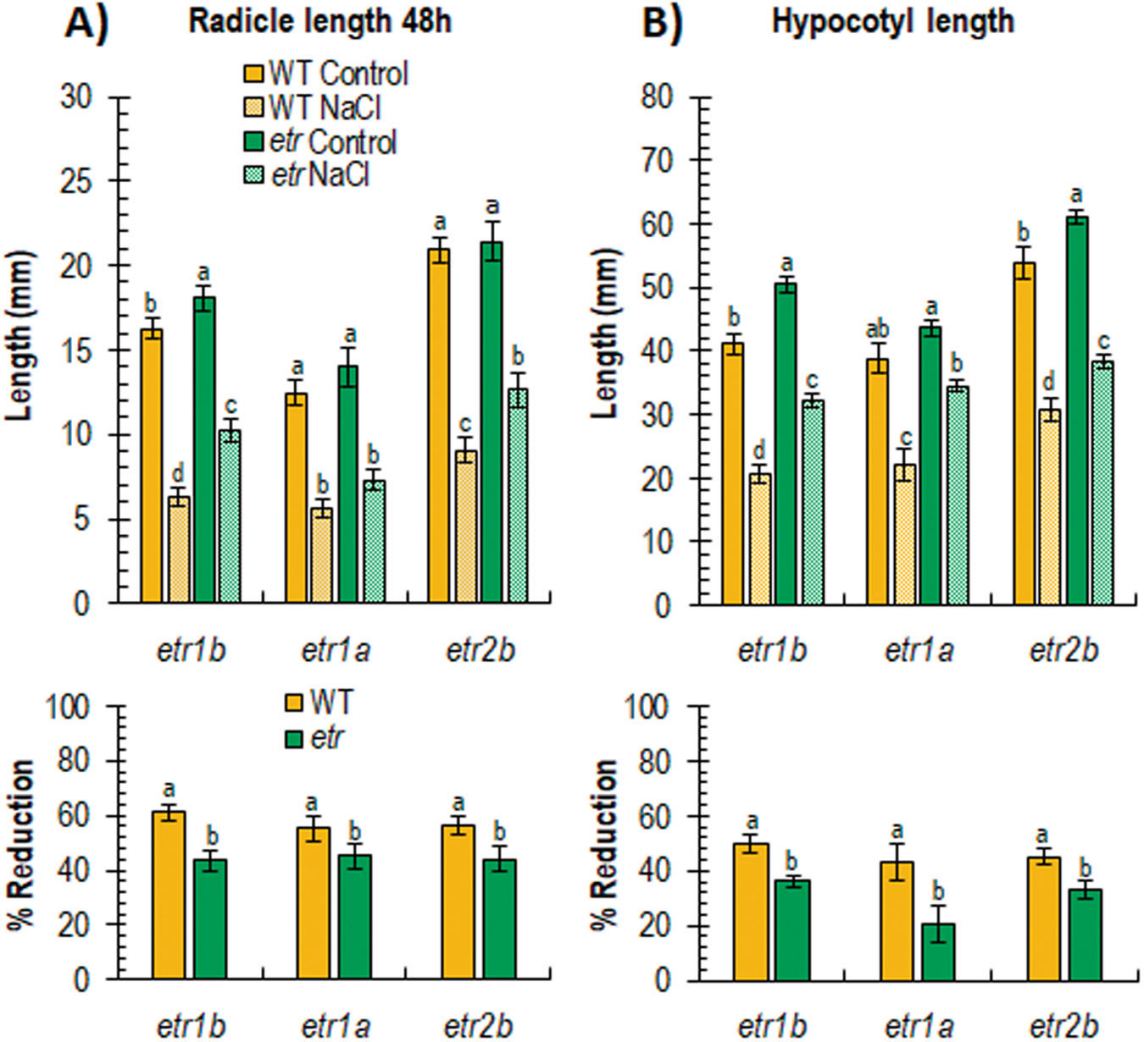
Effect of salt stress on growth parameters of WT and *etr1b*, *etr1a*, and *etr2b* seedlings. **A** Effect of salt stress on radicle length at 48 h. **B** Effect of salt stress on hypocotyl length in seedlings growing in darkness for 72 h. The bottom graphs of each figure show the percentage of reduction of each parameter in response to salt stress in WT and mutant plants with respect to plants of the same genotype growing under control conditions. Different letters indicate statistically significant differences (*P* < 0.05) between samples

Seedling growth was also differentially reduced by salinity in WT and ethylene-insensitive mutants (Fig. 2). Radicle and hypocotyl growth rates were both reduced in response to NaCl treatments in WT and *etr* mutants, but the mutant seedlings were always less affected than WT ones (Fig. 2). When germinated and grown in water, the length of the radicle 48 h after germination was similar in WT and mutants, but the reduction of the radicle length under salt stress conditions was much more noticeable in the WT seedlings (Fig. 2A). The same was true for the length of the hypocotyl 3 days after germination, a parameter that was much more reduced in WT than in *etr* mutants (Fig. 2B). Under salt stress, in fact, WT seedlings reduced the length of their hypocotyls by approximately 50%, while *etr* mutants exhibited a reduction of only 20–35% (Fig. 2A, B).

**Fig. 3:**
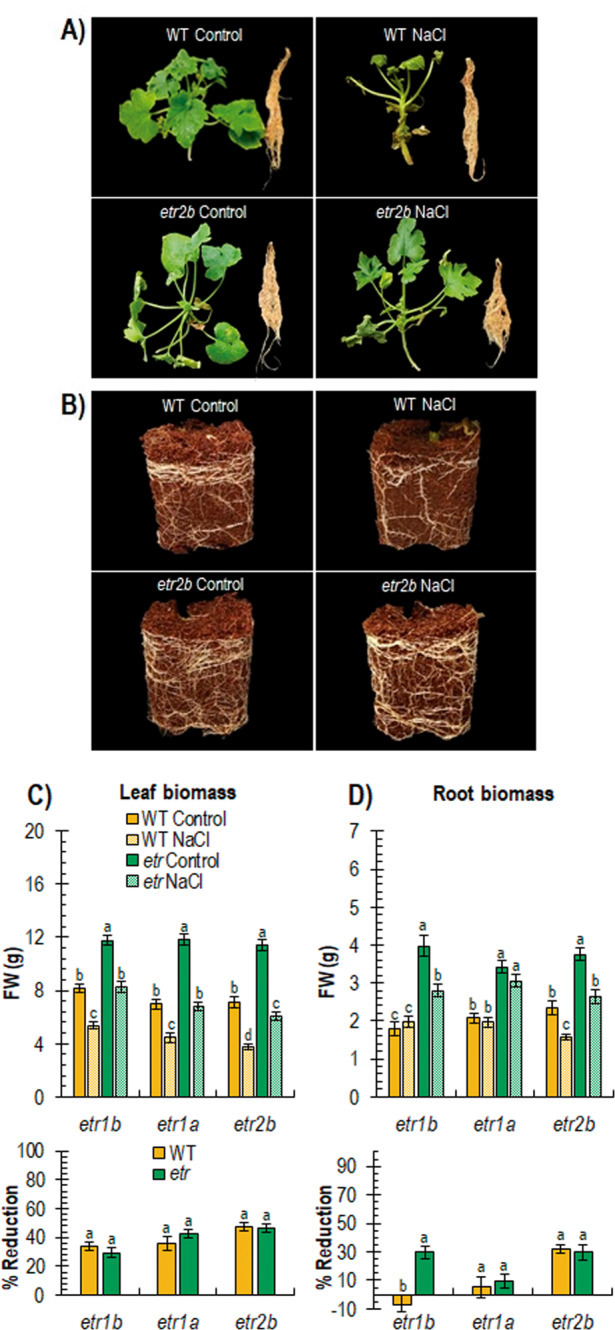
Effect of salt stress on the growth of WT and *etr1b*, *etr1a*, and *etr2b* plants grown for 20 days under control and NaCl conditions. **A** WT and *etr2b* shoots and roots. **B** Root balls of WT and *etr2b* plants. **C**, **D** Effect of salt stress on leaf and root biomass. The bottom graphs of each figure show the percentage of reduction of each parameter in response to salt stress in WT and mutant plants with respect to plants of the same genotype growing under control conditions. Different letters indicate statistically significant differences (*P* < 0.05) between samples

Figure 3A, B shows the effect of salt stress on the root and shoot growth, and root balls of WT and *etr* mutants, 20 days post-germination. Under control conditions, the root and leaf biomass of mutant seedlings was much higher than that of WT, indicating a higher vigour in the three mutant plants (Fig. 3C, D). Although root biomass was decreased considerably under salt stress, that of mutant plants was similar to that of the WT control plants grown in water (Fig. 3C). The biomass of the aerial part of the plant was also significantly higher in the *etr* mutants, and although reduced by the NaCl treatment, the leaf biomass of the mutant plants under salt stress was also higher than that of the WT (Fig. 3D). The reduction in leaf and root biomass in response to salt stress was not significantly different between WT and mutant plants (Fig. 3C, D). These data demonstrate that *etr1b*, *etr1a* and *etr2b* seedlings were more vigorous than those of WT under control and salt conditions, but the responsiveness of WT and mutant plants to salt stress did not significantly differ, at least during the first 20 days of vegetative development.

### Growth and ionic balance of WT and *etr2b* plant in response to salt stress

**Fig. 4:**
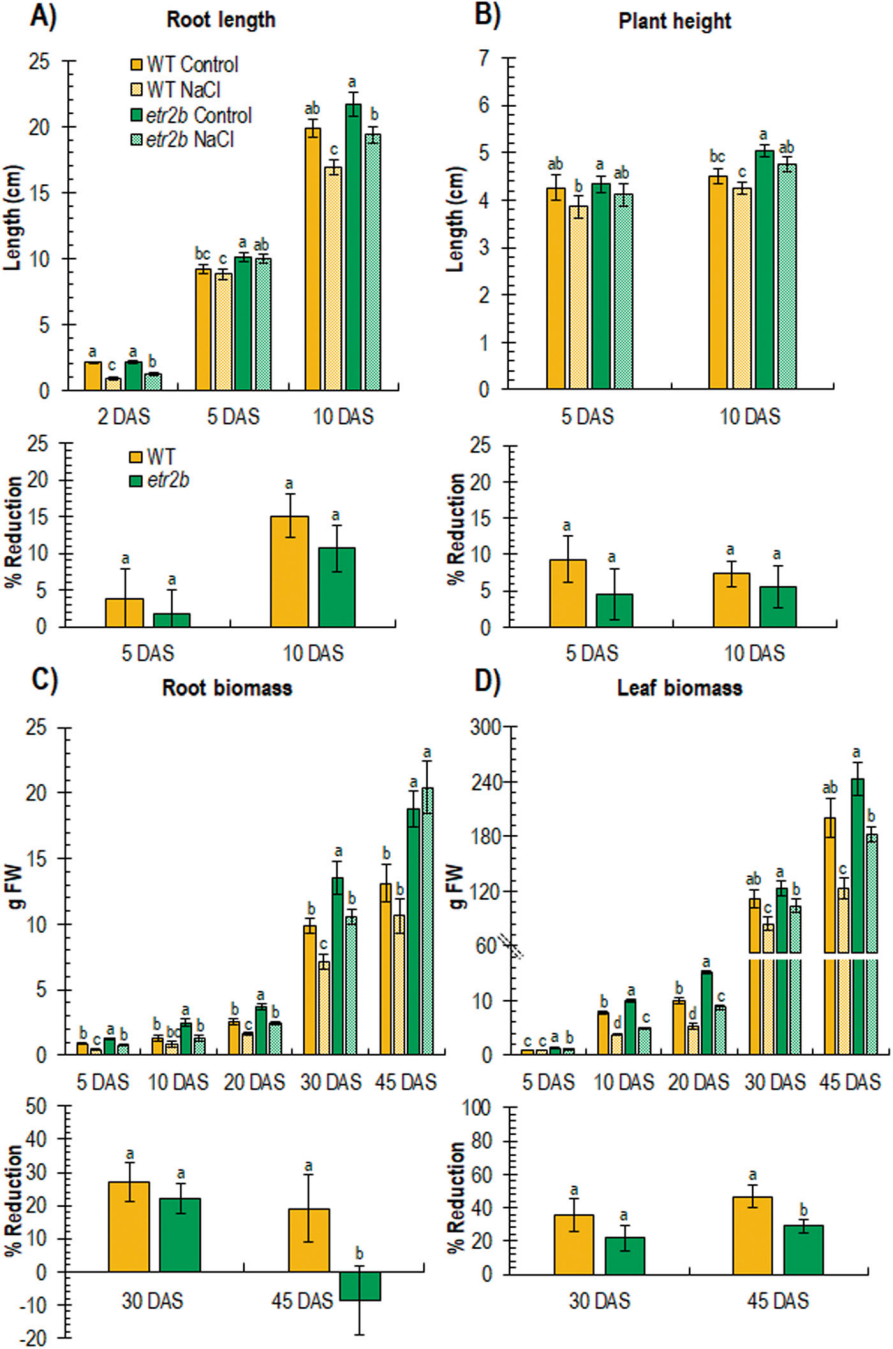
Effect of salt stress on root and leaf development of WT and ethylene receptor *etr2b* mutant of *C. pepo* at different days after sowing (DAS). The graphs at the top in **A**, **B**, **C** and **D** show the growth rates of root length and plant height, as well as root and leaf biomass, in plants growing under control and NaCl conditions. The graphs at the bottom show the percentage of reduction of the same parameters in response to salt stress in WT and mutant plants with respect to plants growing under control conditions. Different letters indicate statistically significant differences (*P* < 0.05) between samples

The separation of WT and *etr2b* offspring (see Materials and Methods) allowed further analyses of this ethylene receptor mutant. WT and *etr2b* plants were grown under control and saline conditions up to 45 days after sowing (DAS) (Fig. 4).

The growth of roots, shoots and leaves was always higher in the mutant (Fig. 4) under both control and salt stress, which confirmed the higher vigour of the ethylene receptor mutants observed in previous experiments, and the higher salt tolerance of the mutant. However, the relative response of WT and mutant plants to salt stress differed throughout plant development. At early stages (5 and 10 DAS) *etr2b* and WT responded similarly to salt stress, reducing both plant height and root length (Fig. 4A, B). The reduction in leaf and root biomass between 5 and 30 DAS was also similar in WT and mutant plants (Fig. 4C, D). At 45 DAS, however, the salt sensitivity of the mutant was significantly lower than that of the WT, with *etr2b* exhibiting a significantly lower percentage of reduction in leaf and root biomass than WT (Fig. 4C, D). These data indicate that *etr2b*, and probably the other two ethylene receptor mutants, have an enhanced tolerance to salt stress not only during germination, but also during plant vegetative development.

Table 1 shows the effect of salinity on the nutrient content of WT and *etr2b* leaves and roots. For most of the nutrients, no significant differences were found between WT and *etr2b* plants in either roots or in leaves. For K and Ca, no differences were identified between WT and *etr2b*, except that the Ca content was slightly lower in the mutant under non-saline conditions. The total N content was reduced in response to salt stress in both WT and mutant leaves, but no difference was detected between the response of the two genotypes. As expected, salt-stressed plants increased their content of the phytotoxic elements Cl^−^ and Na. In leaves of salt-stressed plants, Na accumulated at least 4.5 mg/kg more in WT than in *etr2b*, but Cl^−^ content was found to be similar in the two genotypes. In roots, however, no significant difference was found between WT and *etr2b* for either Na or Cl^−^ (Table 1).

**Table 1:** Content of macronutrients, micronutrients, and phytotoxic elements in WT and etr2b mutant leaves and roots of plants grown under Control and NaCl conditions for a total of 45 days after sowing (DAS)

	Macronutrients (%)						Micronutrients (mg/kg)					Phytotoxic elements (mg/kg)	
	N total	P	K	Ca	Mg	S	Fe	Mn	Cu	Zn	B	Cl^−^	Na
Leaves													
WT control	5.23 a	1.40 a	7.19 a	4.16 ab	0.50 a	0.38 a	187.67 a	202.33 a	5.91 c	77.73 b	131.33 a	45.85 c	1.92 c
WT NaCl	4.54 c	1.20 a	6.85 a	4.06 ab	0.44 a	0.39 a	245.33 a	219.67 a	15.73 a	100.20 a	117.67 a	86.05 a	14.19 a
* etr2b* control	5.08 b	1.54 a	8.24 a	3.72 b	0.43 a	0.40 a	235.00 a	220.33 a	6.69 c	85.23 ab	125.50 a	63.71 b	1.76 c
* etr2b* NaCl	4.56 c	1.18 a	7.23 a	4.50 a	0.46 a	0.38 a	199.67 a	240.33 a	10.10 b	86.80 ab	110.00 a	82.73 a	9.61 b
Root													
WT control	3.23 b	1.66 a	2.00 a	1.47 a	0.17 a	0.28 a	319.00 a	165.67 b	8.29 b	69.23 ab	33.80 ab	7.37 b	7.32 b
WT NaCl	3.19 b	1.32 c	2.40 a	0.81 b	0.16 a	0.33 a	292.00 a	212.50 ab	8.57 ab	59.85 b	30.35 bc	20.78 a	13.92 a
* etr2b* control	3.29 ab	1.58 ab	2.35 a	1.27 ab	0.16 a	0.33 a	312.33 a	178.00 b	9.28 ab	62.77 ab	35.47 a	10.76 b	8.32 b
* etr2b* NaCl	3.47 a	1.41 bc	2.33 a	1.12 ab	0.16 a	0.33 a	289.67 a	232.00 a	10.95 a	76.50 a	30.33 c	18.82 a	12.91 a

Different letters within the same column indicate significant differences between means (*P* < 0.05)

### Comparison of stress metabolites and gene expression in WT and *etr2b* in response to salt stress

**Fig. 5:**
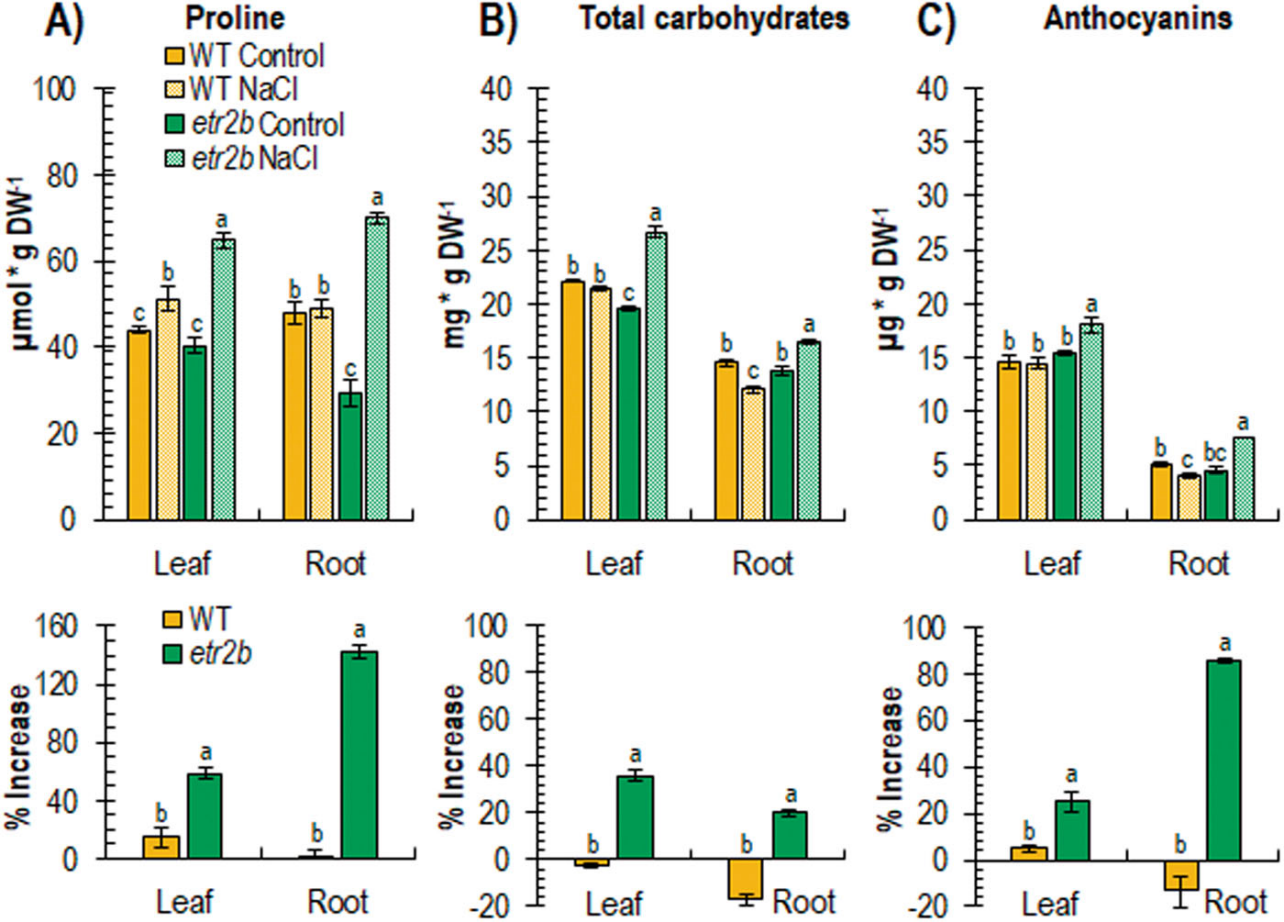
Content of stress metabolites in WT and *etr2b* mutant leaves of plants growing under control and NaCl conditions for a total of 45 days after sowing (DAS). **A**, **B** and **C** shows the content of proline, total carbohydrates and anthocyanins, respectively. The bottom graphs show the increment in metabolite content in response to salt stress in WT and mutant plants. DW, dry weight. Different letters indicate statistically significant differences (*P* < 0.05) between samples

To gain insight into the mechanisms that regulate the enhanced salt tolerance of *etr2b*, the content of some metabolites and the expression of genes related to salt stress in different plant systems were measured. Figure 5 shows the contents of proline, total carbohydrate, and anthocyanin in leaves and roots of WT and mutant plants grown under either control or salinity conditions for 45 days. Under control conditions, most of the assessments were similar in WT and *etr2b* plants, although *etr2b* roots showed a decreased content of proline, and *etr2b* leaves reduced their content in total carbohydrates (Fig. 5). In salt-stressed plants, the response of WT and mutant plants was completely dissimilar. Salt induced the accumulation of proline, total carbohydrates and anthocyanins in both roots and leaves of the mutant plants, but hardly changed their contents in WT in either roots or in leaves (Fig. 5A–C).

The expression of genes associated with abiotic stress tolerance was also compared in WT and *etr2b* plants grown over 45 days under standard and saline stress conditions. Since the *C. pepo* genome is duplicated, we investigated the expression of paralogs from both A and B subgenomes (indicated by the letter A or B at the end of the gene name, respectively). The phylogenetic relationship between *C. pepo* selected genes (Supplementary Table S2) and Arabidopsis homologues with known functions was previously examined for each gene family (Supplementary Fig. S2), thus providing a likely function of the analysed genes in *C. pepo*. In fact, the name that we assigned to each *C. pepo* gene corresponds to the Arabidopsis gene, which had the most conserved protein identity (Supplementary Fig. S2).

**Fig. 6:**
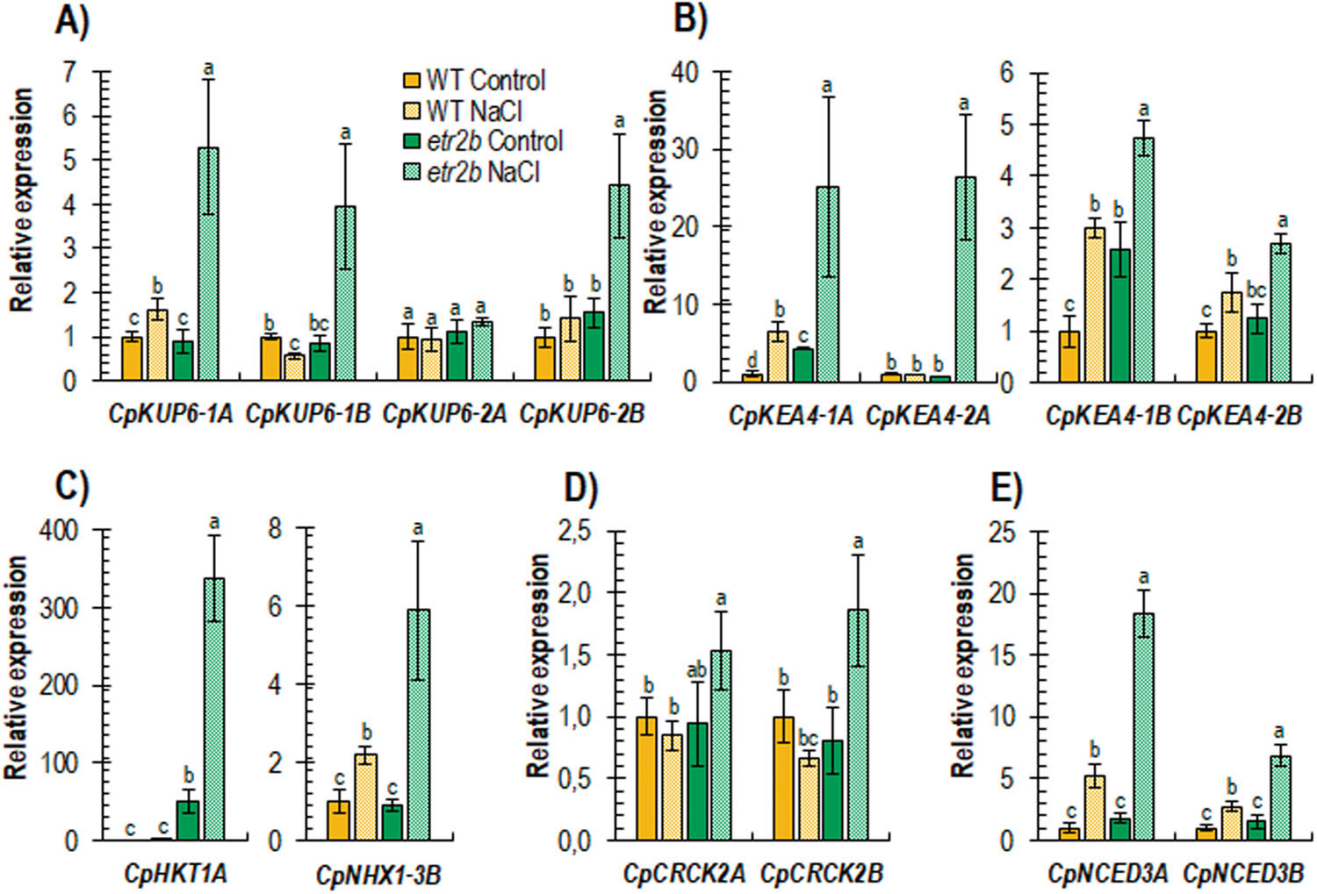
Relative expression of genes encoding for ion transporters, salt stress signalling and ABA biosynthesis in leaves of WT and etr2b plants grown for 45 days under control and NaCl conditions. **A** Potassium transporters CpKUPs. **B** K+/H+ efflux antiporters CpKEAs. **C** Sodium transporter CpHKT1A and Na+/H+ exchanger CpNHX1-3B. **D** Calmodulin-binding receptor-like cytoplasmic kinase, CpCRCKs. **E** 9-cis-epoxycarotenoid dioxygenase CpNCEDs, involved in ABA biosynthesis. In each gene family, the number of each gene corresponds to that of Arabidopsis with the highest identity at the protein sequence level, and the A and B letters at the end of each gene corresponds to paralogs derived from the A and B subgenomes of *C. pepo*, respectively. The relative level of each transcript was assessed by qRT–PCR in three independent replicates and normalised by the ∆∆CT method. Different letters indicate statistically significant differences (*P* < 0.05) between samples

The expression of most of the genes associated with salt tolerance was much more induced in the mutant than in the WT plants, indicating an enhanced response of *etr2b* plants to salt stress (Fig. 6). K^+^ transporter genes, including K^+^ uptake permeases or KUPs (*CpKUP6-1A, CpKUP6-1B and CpKUP6-2A, CpKUP6-2B*), and K^+^/H^+^ efflux antiporters or KEAs (*CpKEA4-1A, CpKEA4-1B* and *CpKEA4-2A, CpKEA4-2B*), with the exception of *CpKUP6-2A*, were upregulated by NaCl in both WT and *etr2b*, but the upregulation in the mutant was between 2 and 25 times higher than in the WT (Fig. 6A, B). Under control conditions, some of them, including, *CpKEA4-1A*, *CpKEA4-1B*, were also more expressed in mutant than in WT plants.

The same is true for genes encoding Na^+^/H^+^ exchanger *CpNHX1-3B* and Na^+^ transporter *CpHKT1A*, which were only upregulated in *etr2b* plants when grown under saline conditions (Fig. 6C). Of particular interest is the *CpHKT1A* gene, whose expression was already highest in non-stressed mutant plants, and was upregulated more than 300-fold in response to salinity in only the *etr2b* mutant (Fig. 6C). Genes involved in abiotic stress signalling pathways, including Ca^2+^ signalling gene Calmodulin-binding receptor-like kinases *CpCRCK2A* and *CpCRCK2B*, and ABA biosynthesis genes *CpNCED3A* and *CpNCED3B*, were also more highly induced in *etr2b* plants than in WT in response to salt stress (Fig. 6D, E).

## Discussion

Ethylene is a key modulator of plant response to salt stress, but its specific role in different plant species and plant developmental stages is unclear^24,27,32^. In Arabidopsis and other plants, including maize and tomato, ethylene positively regulates salt stress tolerance^25,45–47^, however, in other plant species, such as rice and tobacco, ethylene plays a negative role in salinity stress response^24,32^. In this paper, we demonstrate that ethylene is also involved in the salt stress response of *C. pepo*. All of the physiological and molecular data presented in this paper indicate that gain-of-function mutations in three *C. pepo* ethylene receptor genes increase salt stress tolerance at germination and during seedling and plant vegetative development, suggesting that ethylene is a negative regulator of salt tolerance in *C. pepo*.

### ETR receptors modulate salt tolerance response at germination and during seedling and plant vegetative growth

Seed germination is severely affected by salinity, being the first process involved in the stress tolerance response^7^. The phenotypes of loss-of-function mutants in Arabidopsis have demonstrated that ETR1 and EIN4 inhibit, while ETR2 enhances, seed germination under salt stress, and ERS1 and ERS2 have no significant effect on seed germination^36,37,39^. Accordingly, Arabidopsis loss-of-function mutations for ETR1, including *etr1-6* and *etr1-7*, are more tolerant to salt stress and germinate before WT; whereas, gain-of-function mutants for ETR1, including *etr1-1*, *etr1-2* and *etr1-3*, are more sensitive to salt stress and germinate later than WT under salt stress^48–50^. The accelerated germination of the three analysed gain-of-function *etr1b, etr1a*, and *etr2b* of *C. pepo* under salt stress indicate that CpETR1B, CpETR1A, and CpETR2B are positive regulators of squash seed germination under salt stress. If this function is dependent on ethylene and the ethylene signal transduction pathway, ethylene would play a negative role in *C. pepo* salt tolerance, which is similar to what occurs in rice^24^. However, given that in Arabidopsis the function of ETRs in seed germination can take place independently of the canonical ethylene signal transduction pathway, but appears to be mediated by ABA signalling^36,39^, it is also likely that the mechanisms underlying the function of squash ETRs under salt stress may also occur through ABA rather than the ethylene signalling pathway. The fact that the three *etrs* exhibit similar salt stress tolerance, but differ in the magnitude of their ethylene response (*etr1b* showed the most residual responsiveness and *etr2b* the least^43^), suggests that the function of *C. pepo* ETRs on germination under salt stress could take place independently of ethylene. The reduced germination rate of ethylene-insensitive *etr* mutants in water may also be the consequence of an increased biosynthesis or sensitivity to ABA, as found in Arabidopsis ethylene-insensitive mutants *etr1* and *ein2*^51^. Furthermore, the higher induction of the ABA biosynthesis genes *CpNCED3A* and *CpNCED3B* in *etr2b* plants under salt stress also supports the involvement of ABA in the salt tolerance of this mutant. The assessment of ABA sensitivity and ABA biosynthesis of *C. pepo etr* mutants in the presence and the absence of NaCl will provide insight into the interactions between ABA and ethylene signalling cascades during seed germination.

We also observed that the three *C. pepo etr* mutations promote seedling and plant growth, resulting in higher plant height, and higher root and leaf biomass when grown under control standard conditions. This higher vegetative vigour of *etr* mutants was also identified in adult plants^42,43^, indicating that ethylene is a negative regulator throughout the vegetative development cycle of the plant. The stimulating effect of ethylene insensitivity on vegetative growth was also found in Arabidopsis ethylene-insensitive gain-of-function mutants^49,52,53^, although other studies found no differences in total leaf area between WT and the *etr1-1* mutant at earlier stages of vegetative development^54^. The higher constitutive growth and vigour of squash ethylene-insensitive mutants was correlated with their higher salt tolerance during seedling and vegetative plant development. These data contrast with those found in Arabidopsis, in which the higher vegetative growth of the ethylene-insensitive gain-of-function *etr1-1* mutant and the transgenic Arabidopsis plants overexpressing the tobacco ethylene receptor NTHK1 were associated with a higher salt sensitivity, while the reduced seedling growth of the *etr1-7* loss-of-function mutant was associated with greater salt tolerance^49,55^. The reduced ABA sensitivity of *etr1-7* and the enhanced ABA sensitivity of *etr1-1* may account for differences in plant growth and salt-tolerance, as explained for germination^49,51^.

The enhanced salt tolerance of *C. pepo etrs* during seedling and plant vegetative growth could result from the inhibitory role of ethylene receptors in the ethylene signalling pathway^42,43^. However, it is also likely that the vegetative growth regulation of ethylene receptors occurs through the abscisic acid signalling pathway, as has been observed for ETRs and EIN2 in Arabidopsis^49,51,56^. Genes involved in both ABA biosynthesis and intracellular Ca^2+^ signalling pathway were more induced in the mutant than in WT, which indicates that these two signalling pathways can coordinate the tolerance response of *etr2b* to salt stress. ABA is known to control the expression of ion transport genes and the influx of Ca^2+^ in the guard cells that leads to stomata closure limiting water loss in leaves^57,58^, but also a number of ABA responsive genes that are involved in ion homoeostasis and osmotic adjustment^59^.

### Mechanisms of salt tolerance in *C. pepo etr* mutants

A number of physiological and molecular responses, including Na^+^ detoxification, ion homoeostasis, osmotic adjustment and ROS scavenging, have been developed in plants to combat salt stress^7^. The exclusion of Na^+^ in photosynthetic organs is a mechanism that is widely used by salt-tolerant genotypes to maintain vegetative growth while dealing with the high toxicity of these elements in leaves^3^. In our experiments, the leaves of the salt-stressed WT plant accumulated seven times more Na than non-stressed plants, but the leaves of the salt-tolerant *etr2b* only accumulated 5.3 more Na. Given that WT and *etr2b* roots have similar Na content, these data demonstrate a high ability of the salt-tolerant mutant to restrict the transport of Na^+^ from roots to leaves.

The exclusion of Na^+^ and the higher growth rates of *etr2b* plants are likely to be regulated by the induced Na^+^ and K^+^ transporter genes in the leaf. As occurs with Arabidopsis AtNHX1 and AtNHX2 Na^+^/H^+^ antiporters in the tonoplast, the induced *CpNHX1-3B* may be involved in sequestering Na^+^ into the vacuole, thus reducing the content of Na^+^ in the cytoplasm and alleviating osmotic stress^60,61^. They also function as K^+^/H^+^ antiporters to maintain K^+^ homoeostasis^13^. The CpHTK1A transporter is particularly interesting because it was upregulated 300 times more in *etr2b* than in WT. HTKs are high-affinity transporters for both Na^+^ and K^+^, mediating root Na^+^ uptake, Na^+^ unloading from xylem sap, and leaf Na^+^ refluxing to the phloem, which are mechanisms that increase leaf Na^+^ exclusion^62–65^.

Gene expression data also suggest a positive role of the K^+^ transporters KEAs and KUPs in combating salt stress. The Arabidopsis KEAs are K^+^/H^+^ antiporters that mediate pH and K^+^ homoeostasis in the inner and thylakoid membranes of chloroplast (KEA1, KEA2, and KEA3) or in endomembrane compartments (KEA4, KEA5, and KEA6)^66,67^. The *C. pepo KEAs* genes that were highly induced in *etr2b* under salt stress are highly homologous to the second clade. KUP/HAK/KT, on the other hand, is a large family of high-affinity K^+^ transporters that function in potassium acquisition and translocation from roots to shoots^68,69^, facilitate K^+^ efflux from the vacuole to regulate osmotic adjustment, and some of them are involved in plant growth and development^58,68,70^. The *C. pepo* KUPs that were upregulated in salt-stressed *etr2b* leaf have a higher homology with Arabidopsis KUP6, an ABA responsive K^+^ subfamily transporter that has a key role in osmotic adjustment and K^+^ homoeostasis of guard cells^58^.

*C. pepo etr2b* plants induced the accumulation of metabolites, such as proline, total sugars (glucose, fructose, sucrose, and trehalose) and anthocyanins at both the roots and shoots under salt stress, which demonstrates that ethylene receptors and the subsequent ethylene or ABA signal transduction pathways are mediating the production of these osmolytes and therefore the osmotic adjustment of cells under salt stress^71^. These osmolytes can lower osmotic potential^72^, but can also act as stabilisers of proteins and cell components against ion toxicity and NaCl-induced oxidative damage^71,73^. Proline is perhaps the main salinity-related osmolyte, and is considered a biochemical marker of salt stress^74^. Exogenous proline treatments and transgenics plants with enhanced production of proline are more tolerant to salt, while mutants that are deficient in proline exhibited a limited growth and development under salt stress^75,76^. The biosynthesis of proline and other osmolytes is induced by ABA in different systems^77^, suggesting again that the enhanced response of mutant ethylene receptors of *C. pepo* to salt stress is likely mediated by ABA.

## Materials and methods

### Plant material

The ethylene receptor mutants analysed in this study, *etr1b*, *etr1a* and *etr2b*, were selected from a high-throughput screening of a *Cucurbita pepo* mutant collection by using the triple response of etiolated seedlings to ethylene^41^. In addition to their reduced triple response to ethylene, the three mutations convert female into hermaphrodite or male flowers, reducing or preventing self-fertilisation^42,43^. The mutants were therefore maintained in BC_2_S_1_ segregating generations, obtained by crossing each mutant twice or more times with the background genotype MUC16, and then selfed. The mutations affect *CpETR1B*, *CpETR1A*, and *CpETR2B* genes; thus, the WT and mutant plants in segregating populations were selected by detecting the WT and *etr1b, etr1a*, and *etr2b* alleles using real-time PCR with TaqMan probes^42,43^. DNA was isolated from the cotyledon of seedlings after the development of the first true leaf (5 or 7 days after sowing, DAS) by using the CTAB protocol. The multiplex PCRs were done using the Bioline SensiFAST™ Probe No-ROX Kit, a set of forward and reverse primers amplifying the polymorphic sequence, and two allele-specific probes descriptive of the SNP of interest. The WT probe was labelled with FAM dye, while the mutant probe was labelled with HEX reporter dye. BHQ1 quencher molecule was used in both probes (Supplementary Table S1).

### Seed germination under salinity stress

Seed germination of WT and *etr1b*, *etr1a*, and *etr2b* was tested under salinity stress. Seeds were sterilised with a 5% sodium hypochlorite solution for 10 min and rinsed in distilled water three times, before being incubated in 50 ml Falcon tubes containing 25 ml of distilled water (control) or 100 mM NaCl for 12 h at 25 °C in darkness under continuous shaking. After the imbibition, the seeds were transferred to a dispositive designed to study seed germination (Supplementary Fig. S1). Seeds were placed in a foam strip between two pieces of filter paper and two panes of glass of 12 × 20 cm. This “sandwich glass” was secured with two clips and situated vertically in a recipient with water (control) or 100 mM NaCl solution for seeds to germinate and grow vertically. The sandwich glass with seeds was then incubated in a growth chamber in darkness at 24 °C and 80% RH for 55 h. Three-hundred BC_2_S_1_ seeds, segregating for each *etr* mutant, were germinated and grown using both water and salt in four independent experiments.

The germinated seeds were recorded every 2 h for 55 h through digital images that were processed using ImageJ^®^. Seeds were considered germinated when the seed coat was broken and primary root protrusion was visible (>1 mm). Germination initiation, time of germination at 50% of seeds, and average germination time were determined according to procedures described by Ranal and De Santana^78^. Root elongation from both WT and *ert* mutants was assessed from seedling images at 48 h of initiating germination.

### Seedling and plantlets growth under salinity stress

After germination, seeds were transplanted into 54 seedling trays filled with a mixture of perlite and coconut fibre (20–80%), a substrate with low-cation exchange capacity. 150 seeds of each genotype (WT/WT and *etr*/*etr*), 75 germinated under salt stress and 75 germinated in water, were distributed in three independent experiments. Trays were incubated in a growth chamber in darkness at 24 °C and 80% RH for 72 h, and hypocotyl elongation was assessed in all plants. Control seedlings were irrigated with a nutritive Hoogland solution with a conductivity of 2 dS/m; whereas, for those subjected to salt stress, the nutritive solution was supplemented with 35 mM of NaCl, which increased its conductivity to 5 dS/m.

Seedlings of each segregating population were then genotyped with Taqman probes, and 72 WT/WT and 72 *etr*/*etr* plants from each mutant family were transplanted into 1 l pots containing the same substrate as previously, and grown for 20 additional days at 24 °C under long-day photoperiod (16 h light/8 h dark) and 70% RH in three independent experiments. Half of the plants (36) continued to be irrigated with the standard nutritive solution as previously, while the other half (36) were supplemented with 35 mM of NaCl. Leaf and root biomass were compared between each WT and *ert* mutant grown under both control and salinity conditions.

### Vegetative growth of WT and *etr2b* under salinity stress

Although *etr* mutations affect female fertility and prevent selfing^42,43^, we were able to pollinate the mutant flowers several days prior to anthesis, thus forcing self-fertilisation of the mutant plants and obtaining 100% mutant offspring. This was only achieved in the *etr2b* mutant, which allowed the evaluation of a higher number of plants implementing the analysis of growth parameters in additional plant developmental stages, as well as biochemical and gene expression studies in this mutant. In this mutant family, separated WT and *ert2b* plants were cultivated for up to 45 days under either control or salt conditions following the protocol described in the previous section. The development of different growth parameters, including root length, plant height, and root and leaf fresh and dry weight, were compared between WT and *ert2b* at 5, 10, 20, 30, and 45 DAS. Three independent replicates of ten plants each were analysed for each genotype and irrigation conditions at each developmental stage. At 45 DAS, plants were also used to analyse the effect of *etr2b* mutation on the content in micro- and macro-elements, the accumulation of stress metabolites, and the relative expression of stress-related genes.

### Evaluation of stress-associated metabolites in WT and *etr2b* plants

The concentration of different stress metabolites, including proline, total carbohydrates and anthocyanins, was assessed in dry leaves and dry roots of WT and *etr2b* plants at 45 DAS under control and salinity stress conditions. All determinations were carried out in triplicate, each containing plant material from four plants.

Proline was determined through the ninhydrin method^79^ with minor modifications. 100 mg of dry sample was incubated in a 2 ml of ethanol 60% at 4 °C for 12 h. 0.5 ml of this solution was then mixed with 1 ml of ninhydrin 1%, dissolved in 60% acetic acid, and incubated at 95 °C for 20 min at room temperature. Proline concentration was finally determined by spectrophotometry at 520 nm, and expressed as µmol/g DW. Total carbohydrates concentration was assessed by the phenol-sulphuric method^80^ with minor modifications. 100 mg of dry sample was incubated in 5 ml of ethanol 80% at 80 °C for 1 h, and 1 ml of this solution was then mixed with 1 ml of a solution of phenol 5% and 5 ml of sulphuric acid 95–97%. Total carbohydrates were determined at 490 nm, and expressed as mg/g DW. Anthocyanin content was measured according to Mancinelli^81^. 100 mg of dry sample was incubated at 4 °C for 12 h in 3 ml of a solution of ethanol acidified with 1% of HCl 37%. The spectrophotometry measurements were done at 530 and 657 nm, and the concentrations expressed as µg/g DW. All spectrophotometric readings were performed on 96-well microplates using the BioTek® UV-Visible Epoch™ spectrophotometer.

### Comparison of micro- and macro-elements in WT and *etr2b* plants

Micro- and macro-elements were measured in 5 g of dry leaves and roots coming from the same three samples for each genotype and salinity condition used in the determination of stress metabolites. The elemental measurements were carried out according to the standard protocols dictated by the International Organisation for Standardisation (ISO) (https://www.iso.org/home.html). Total nitrogen was measured through elemental analysis (ISO-13878), chloride was determined by fragmented flow analysis (ISO-15682), and the rest of macro- and micro-nutrients studied (phosphorus, potassium, calcium, magnesium, sulphur, iron, manganese, copper, zinc, boron, molybdenum, and sodium) were assessed by ICP-OES Spectrophotometry (ISO-11885).

### Assessment of gene expression by qRT–PCR in WT and *etr2b* plant

The relative expression of different salt-stress-associated genes was assessed by quantitative reverse transcription (qRT)–PCR in WT and *etr2b* plants grown under control and salt conditions for 45 DAS. The analysis was performed in three biological replicates for each genotype and growing condition, each one derived from a pool of leaves from four plants. Total RNA was isolated from 1 g of leaves according to the protocol of the GeneJET Plant RNA Purification Kit (Thermo Fisher). RNA was reverted to cDNA with the ADNc RevertAid™ Kit (Thermo Fisher). The qRT–PCR was performed in 10 μl total volume with 1×Top Green qPCR Super Mix (BioRad) in the CFX96 Touch Real-Time PCR Detection System Thermocycler (BioRad). The gene expression values were calculated using the 2^−ΔΔCT^ method^82^. EF1α was used as the internal reference gene. Supplementary Table S2 shows the primers used for qRT–PCR reactions in each analysed gene.

### Phylogenetic analysis

MEGA 10 software^83^ was used to establish the phylogenetic relationships between *C. pepo* and *Arabidopsis thaliana* genes encoding for Na^+^ and K^+^ membrane transporters (KUPs, KEAs, NHXs, and HKTs), abscisic acid biosynthesis enzymes (NCEDs), and Calmodulin-binding receptor-like cytoplasmic kinases (CRCKs). Phylogenetic trees were performed using the Maximum Likelihood method based on the Poisson correction model, with 2000 bootstrap replicates. The protein sequences and information were obtained from the Arabidopsis Information Resource (https://www.arabidopsis.org/) and the Cucurbit Genomic Database (http://cucurbitgenomics.org/).

### Statistical analysis

Data were analysed for multiple comparisons by analysis of variance (ANOVA) using the statistical software Statgraphic Centurion XVIII. Differences between genotypes and treatments were separated by the least significant difference (LSD) at a significance level of *P* ≤ 0.05.

## Supplementary Information

Supplementary Information
