# Supplementary material for: Involvement of ethylene receptors in the salt tolerance response of Cucurbita pepo
Source: Hortic Res. 2021 Apr 1;8:73. doi: 10.1038/s41438-021-00508-z (PMC8012379; doi:10.1038/s41438-021-00508-z)
Supplement: Supplementary file 1 — Supplementary Information [file 41438_2021_508_MOESM1_ESM.docx]

| **Supplementary Table S1**. Primers and TaqMan probes used for genotyping *etr1a*, *etr1b* and *etr2b* mutations. | | |
| --- | --- | --- |
| **etr1a family** | **Sequence** | **Tm (^o^C)** |
| **Forward** | GTTCAGTTTGGTGCTTTCAT | 54.3 |
| **Reverse** | ACAAGCATAAGCGCAGTC | 53.8 |
| **Probe C** | FAM — TAACCG**C**TGTGGTATCGTGTGC — BHQ1 | 64.2 |
| **Probe T** | HEX— TAACCG**T**TGTGGTATCGTGTGC —BHQ1 | 64.2 |
| **etr1b family** |  |  |
| **Forward** | GGTTCTTGTTCAGTTTGGTG | 56.4 |
| **Reverse** | ATAATATGTACAAGCATAAGGGCA | 58.3 |
| **Probe C** | FAM—TTTAA**C**CGCTGTGGTATCGTGTGCAA—BHQ1 | 66.2 |
| **Probe T** | HEX—TTTAA**T**CGCTGTGGTATCGTGTGCAA—BHQ1 | 64.2 |
| **etr2b family** |  |  |
| **Forward** | TAGCTTGGCTTGCCATCA | 53.8 |
| **Reverse** | TCTTGGAGTAACCAGGAAC | 55.0 |
| **Probe C** | FAM —AGCTGGGACT**C**CTCCAGAAG— BHQ1 | 62.5 |
| **Probe T** | HEX—AGCTGGGACT**T**CTCCAGAAG—BHQ1 | 60.5 |

| **Supplementary Table S2**. List of primer sequences used for qRT-PCR analysis. | | | |
| --- | --- | --- | --- |
| **Genes** | **Forward Primer** | **Reverse Primer** | **Transcript Name**  **(Cucurbit Genetic Database)** |
| ***CpKUP6-1A*** | GCTCCCTTCCAAATCATCAA | CGTTCCGTAATGTTGCAATG | Cp4.1LG13g08840 |
| ***CpKUP6-1B*** | TTCATCCGATCCGAGAAAAC | GATCGGCAAAGAACACGATT | Cp4.1LG01g23230 |
| ***CpKUP6-2A*** | AAACGGGGAGGCTGTTTAGT | TGACCCCCAATAGCACTCTC | Cp4.1LG03g07680 |
| ***CpKUP6-2B*** | CGAGTTCGAGAAGGACTTGG | ACATCCATTTGTGGGCTCTC | Cp4.1LG04g05290 |
| ***CpKEA4-1A*** | TCAACCCACTCGGAGTCAC | AAATGGAAAGGGAAGGGAAA | Cp4.1LG18g03650 |
| ***CpKEA4-1B*** | ATTGGACCCGGAGGTTTAAG | CCTCCTAGAACAGCCACTGC | Cp4.1LG04g10710 |
| ***CpKEA4-2A*** | CCCCTACCGTCTCTCTTCCAC | AAGATGAAAATGCGGACAGG | Cp4.1LG11g00130 |
| ***CpKEA4-2B*** | ATCGGCACCCTTATCTTGC | GCAGAATGCCACTGATGCTA | Cp4.1LG07g06210 |
| ***CpHKT1A*** | CCTAAAACTCGCTCCGACTG | TTGGCACAAACCCACAACTA | Cp4.1LG18g06540 |
| ***CpNHK1-3B*** | CACTCAACTGATCGGGAGGT | CAAATGAGAGCGTTGCAAAA | Cp4.1LG12g05410 |
| ***CpCRCK2A*** | CCTCCTTTCCATAGCTTTGAAGT | CGGGTCGGGTGGACTATTAT | Cp4.1LG16g06470 |
| ***CpCRCK2B*** | GCTCATGCTATCACCTATCTTCA | TAACTTGGGTCGAGACATGC | Cp4.1LG05g09620 |
| ***CpNCED3A*** | CTTCGTGGGTCAAATCTGGT | GACACTGAAGGAGGCGAAAC | Cp4.1LG14g03280 |
| ***CpNCED3B*** | CTGCTCCCTCCGTGTCTTC | ACAAGTGATGACCGGAAACC | Cp4.1LG01g00800 |
| **EF1α** | CGTCAAGAAGAAATAAGCCA | CTACTACGAGAGAGAGAGCCG |  |


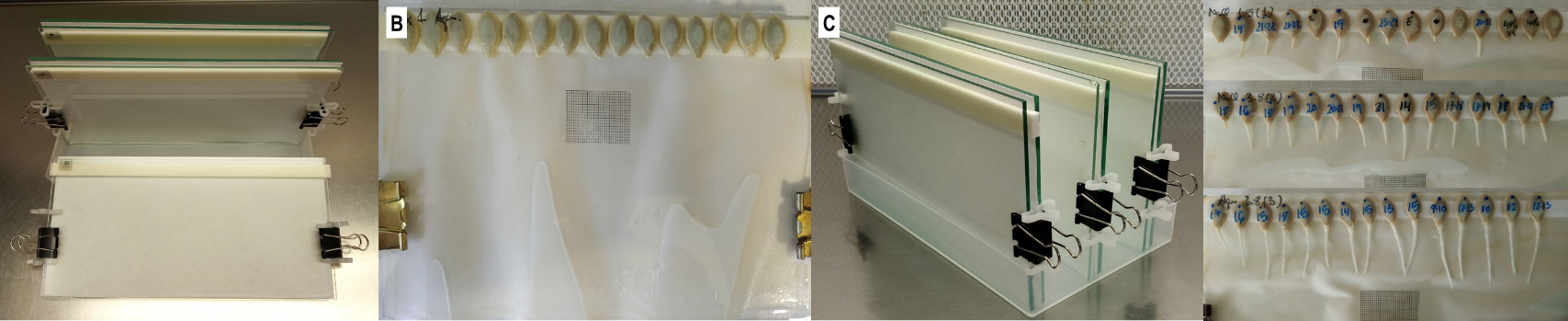


**Supplementary Figure S1. A**. Device designed to study *C. pepo* seed germination between two sandwich glasses. **B**. Seeds were put in a foam strip placed between two pieces of filter papers and two panes of glass of 12 x 20 cm, and secured with two paperclips. **C**. The device is situated vertically in a recipient with treatment solutions for seeds to germinate and grow vertically and placed in an environmentally controlled growth chamber. **D**. Germinated seeds at different times.


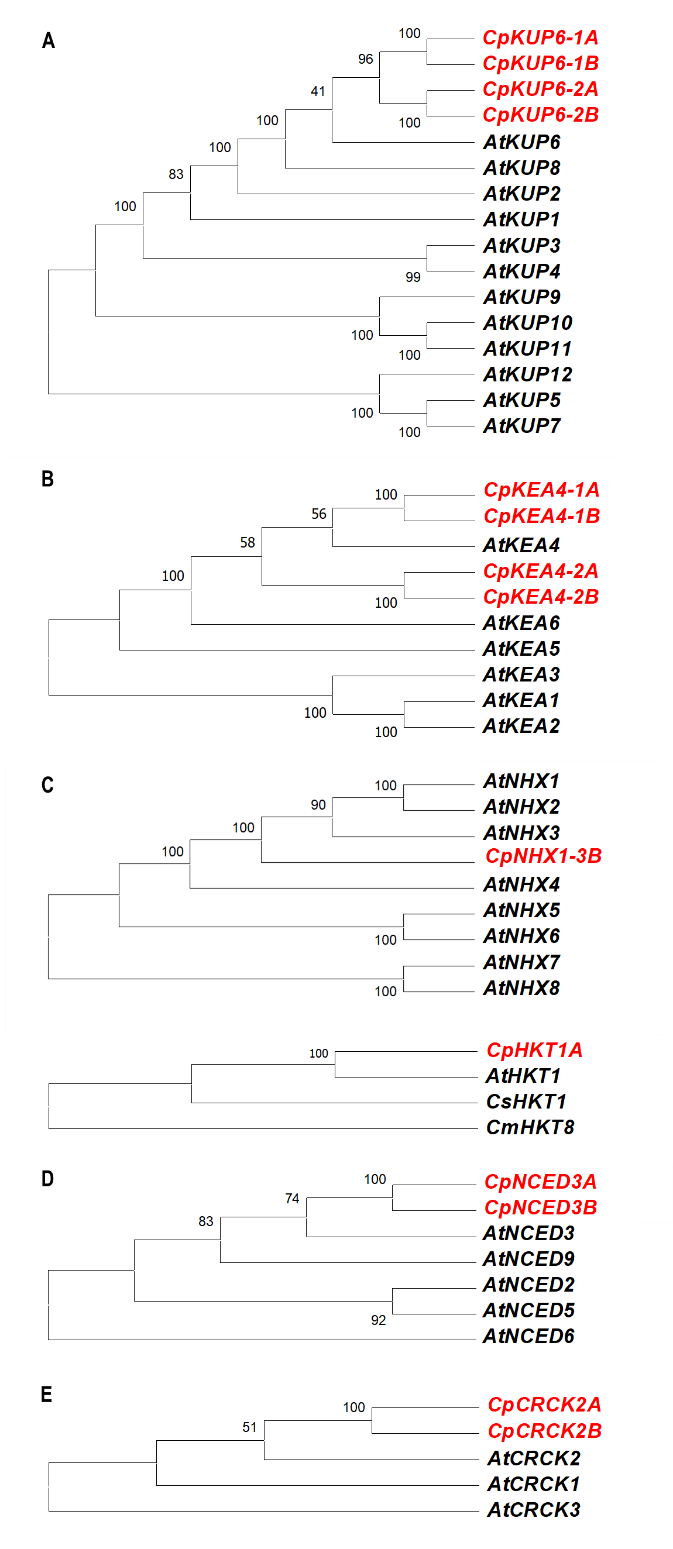


**Supplementary Figure S2**. Phylogenetic relationships for different gene families in *Cucurbita pepo* and *Arabidopsis*. **A**. Potassium transporters (*KUPs*). **B**. K^+^/H^+^ antiporters (*KEAs*). **C**. Na^+^ transporter (*HKTs*) and Na^+^/H^+^ exchangers (*NHXs*). In this case, *Cucumis sativus* and *Cucumis melo HKT* genes were also included. **D**. 9-cis-epoxycarotenoid dioxygenase (*NCEDs*). **E**. Calmodulin-binding receptor-like cytoplasmic kinase (*CRCKs*).
